# Supplementary material for: ROS-Induced DNA-Damage and Autophagy in Oral Squamous Cell Carcinoma by Usnea barbata Oil Extract—An In Vitro Study
Source: Int J Mol Sci. 2022 Nov 27;23(23):14836. doi: 10.3390/ijms232314836 (PMC9738295; doi:10.3390/ijms232314836)
Supplement: Supplementary file 1 [file ijms-23-14836-s001.zip › Supplementary Material Figures S1-S8 and Table S1 .pdf]

International Journal of *Molecular Sciences*

MDPI

Article

ROS-Induced DNA-Damage and Autophagy in Oral Squamous Cell Carcinoma by *Usnea barbata* Oil Extract – an *In Vitro* Study – Supplementary Material

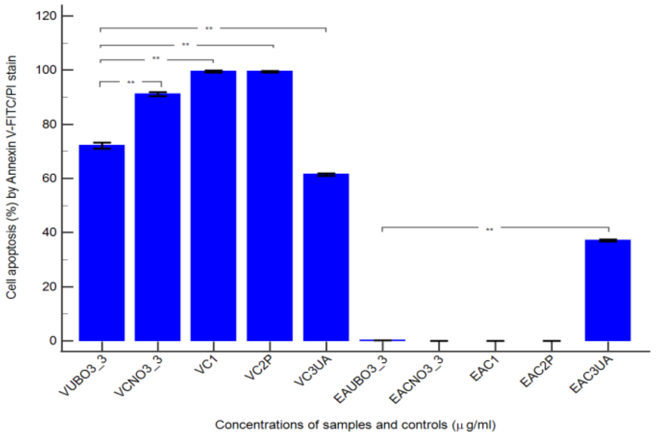

(a)

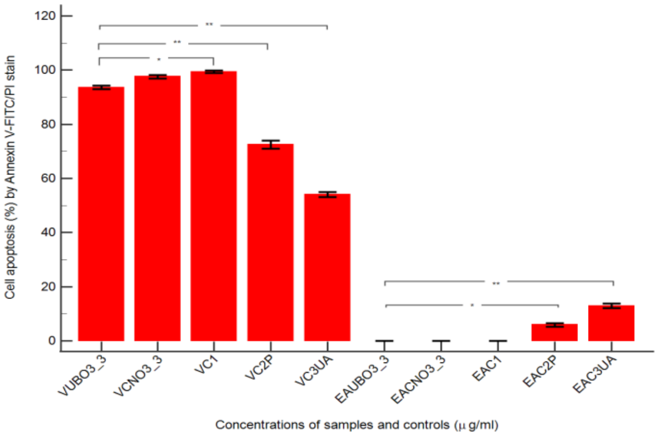

(b)

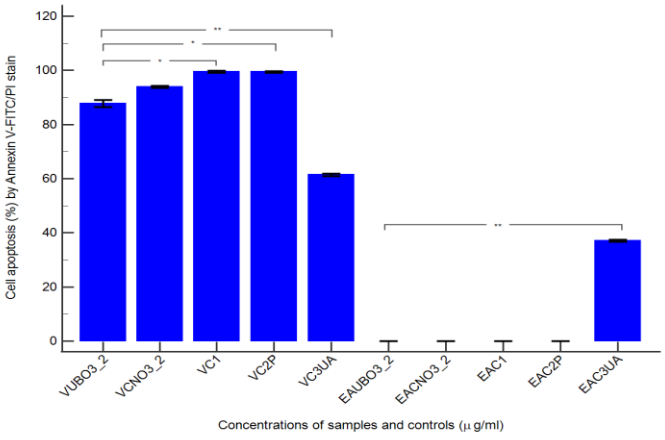

(c)

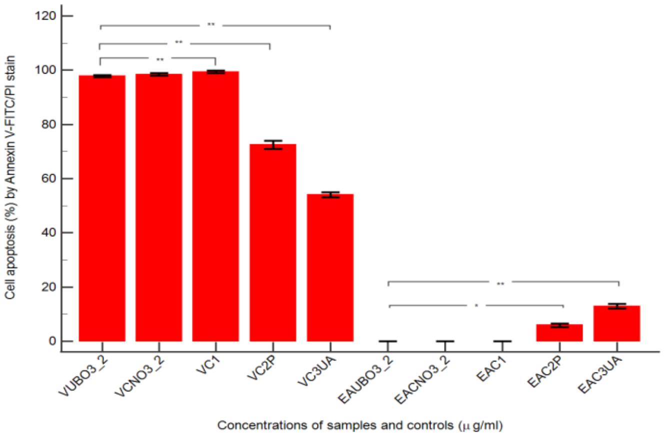

(d)

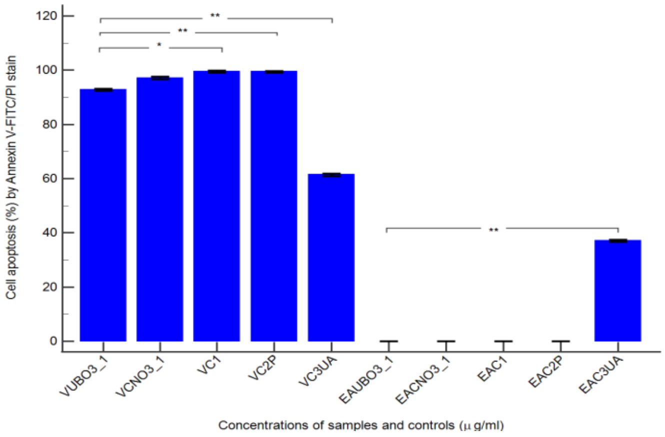

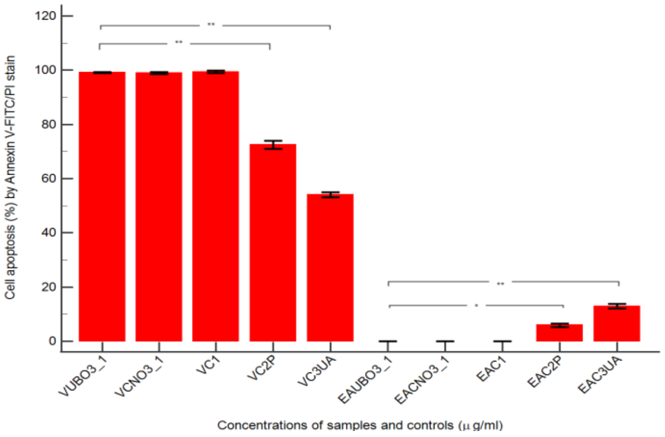

(d) **(e)**

Figure S1. Statistical analysis of cell apoptosis in blood cells (**a, c, and w**) and CLS-354-tumor cells (**b, d, and e**) after 24 h exposure to UBO and CNO 3:3 (**a** and **b**), 3:2 (**c** and **d**) and 3:1 (**e** and **f**) * $p < 0.05$ together with ** $p \leq 0.01$ indicates statistically significant differences between controls and samples. C1—1% DMSO (negative control), C2P—5% Poloxamer 407 (positive control), C3UA—usnic acid (positive control), V—viability, EA—early apoptosis, LA—late apoptosis, N—necrosis. [3:3; 3:2; 3:1]—culture medium/sample ratio (*v*/*v*).

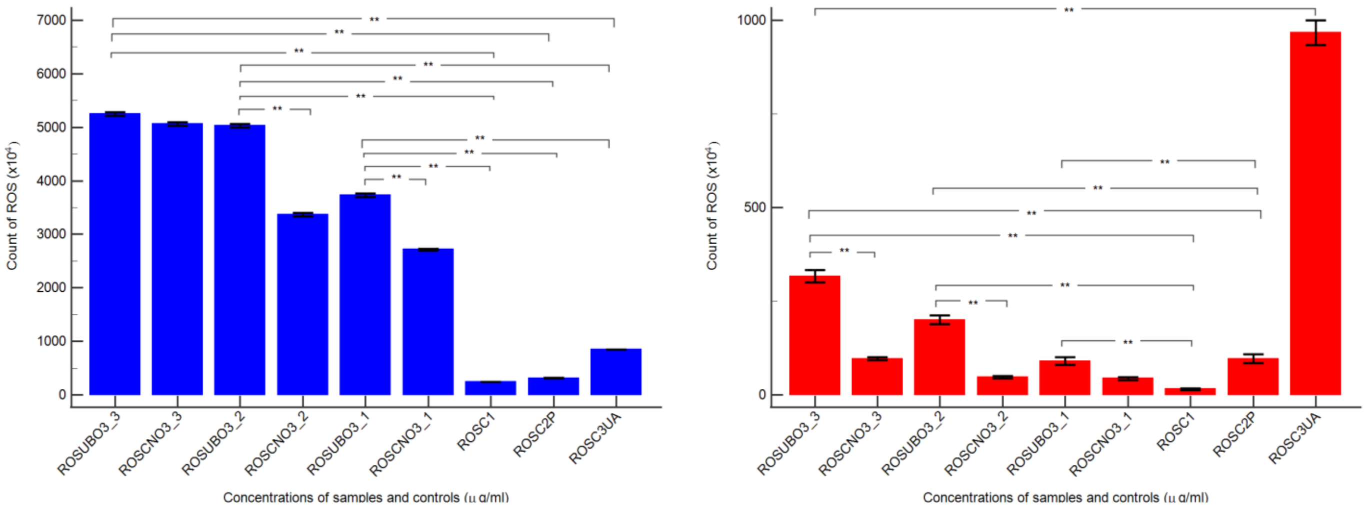

(a) **(b)**

Figure S2. Statistical analysis of total ROS activity expressed as a count of ROS $\times 10^4$ in blood cells (**a**) and CLS-354 tumor cells (**b**) after 24 h exposure to UBO and CNO. **$p \leq 0.01$ indicates statistically significant differences between controls and samples. C1—1% DMSO (negative control), C2P—5% Poloxamer 407 (positive control), C3UA—usnic acid (positive control), ROS—reactive oxygen species. [3:3; 3:2; 3:1]—culture medium/sample ratio (*v*/*v*).

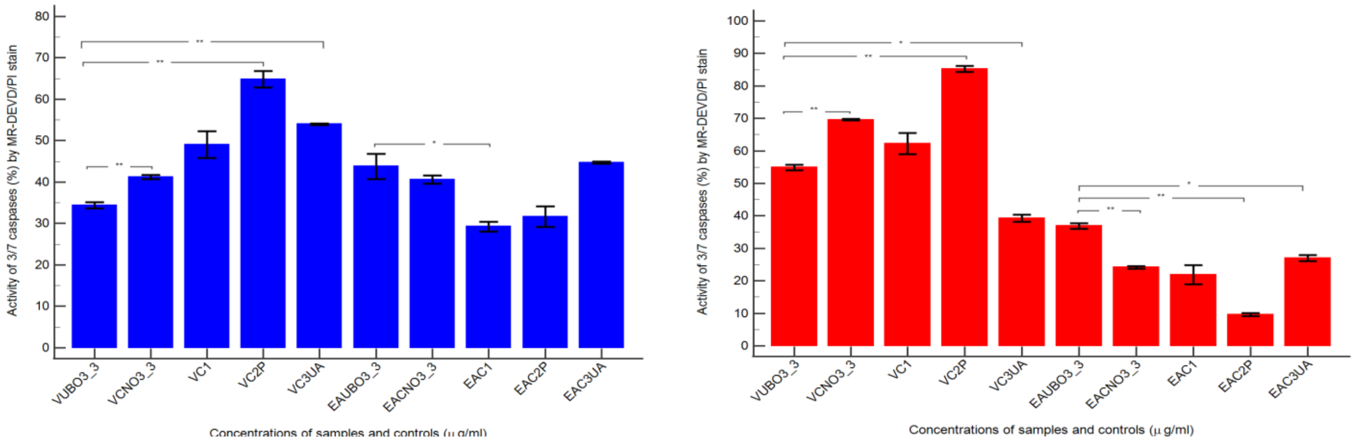

(a) **(b)**

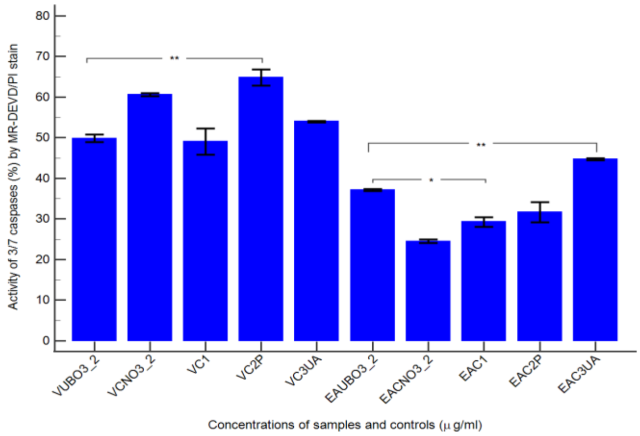

(c)

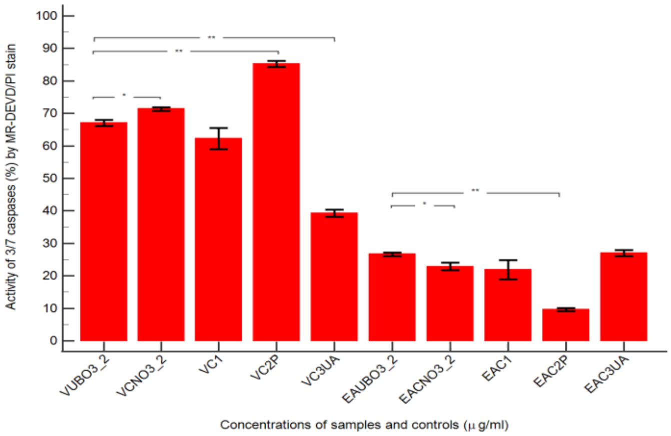

(d)

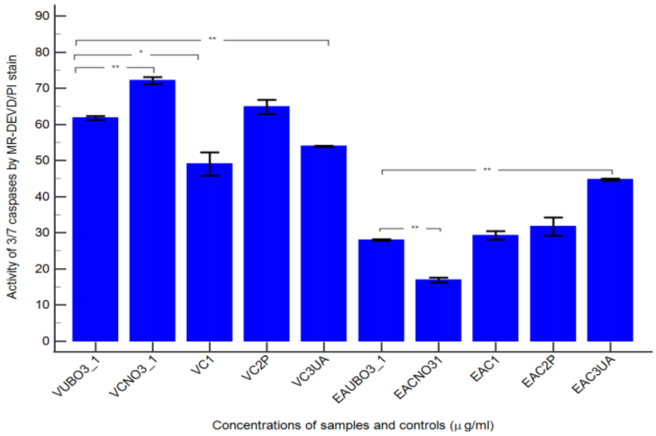

(e)

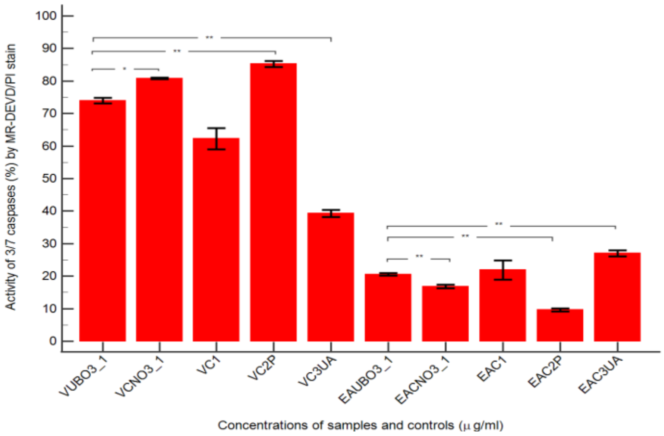

(f)

Figure S3. Statistical analysis of the enzymatic activity of caspase 3/7 in blood cells (**a, c, and e**) and CLS-354 tumor cells (**b, d, and f**) after 24 h exposure to UBO and CNO in different concentrations: 3:3 (**a** and **b**) 3:2 (**c** and **d**) 3:1 (**e** and **f**) * $p < 0.05$ together with ** $p \leq 0.01$ indicate statistically significant differences between controls and samples. C1—1% DMSO (negative control), C2P—5% Poloxamer 407 (positive control), C3UA—usnic acid (positive control); V—viability; EA—early apoptosis; LA—late apoptosis; N—necrosis. [3:3; 3:2; 3:1]—culture medium/sample ratio (*v*/*v*).

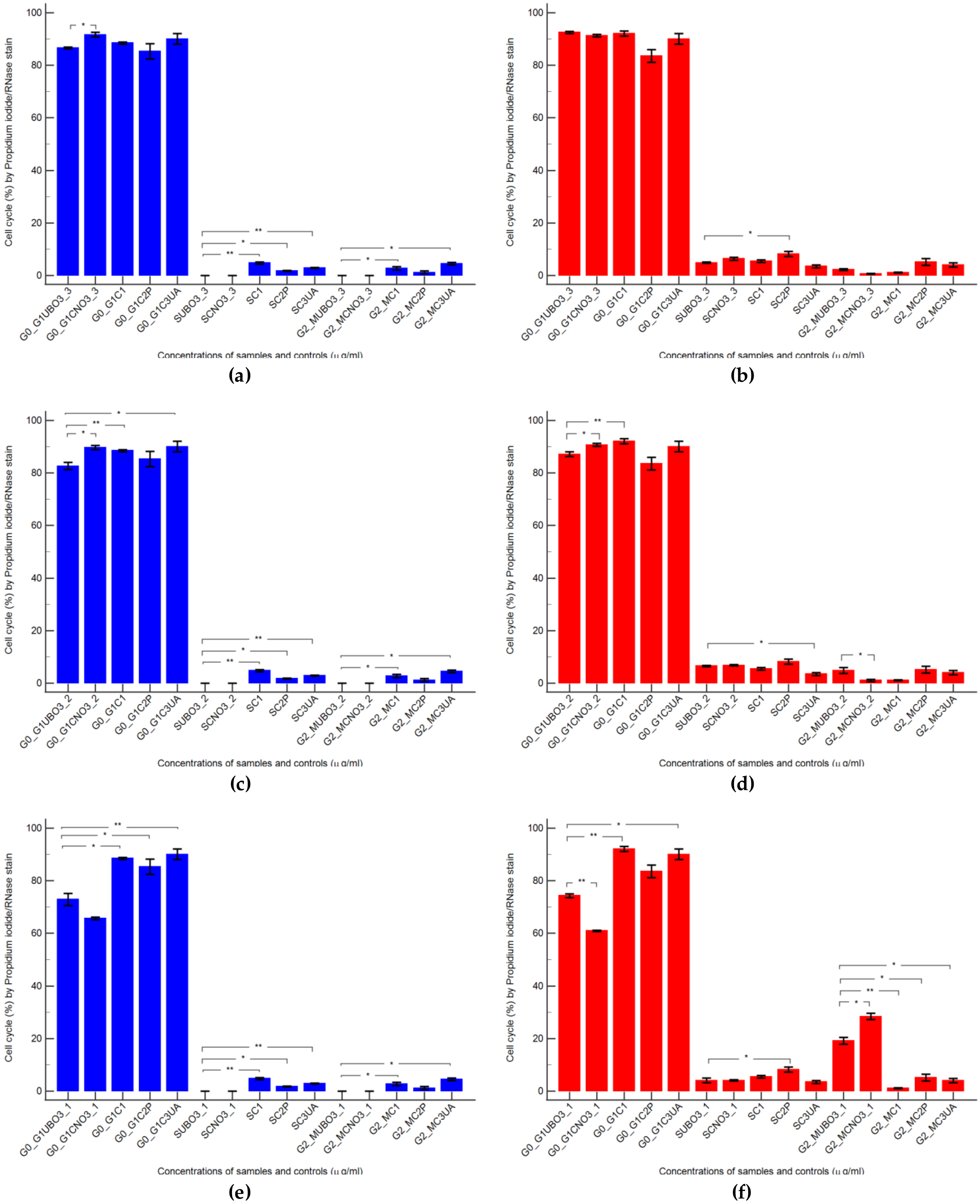

Figure S4. Statistical analysis of cell cycle in blood cells (**a, c, and d**) and CLS-354-tumor cells (**b, d, and e**) after 24 ore exposure to UBO and CNO in different concentrations: 3:3 (**a** and **b**), 3:2 (**c** and **d**) and 3:1 (**e** and **f**). *$p < 0.05$ together with ** $p \leq 0.01$ indicate statistically significant differences between controls and samples. C1—1% DMSO (negative control), C2P—5% Poloxamer 407 (positive

control), C3UA—usnic acid (positive control), AP—cell apoptosis (DNA fragmentation, subG0/G1), S—DNA synthesis; [3:3; 3:2; 3:1]—culture medium/sample ratio (*v*/*v*).

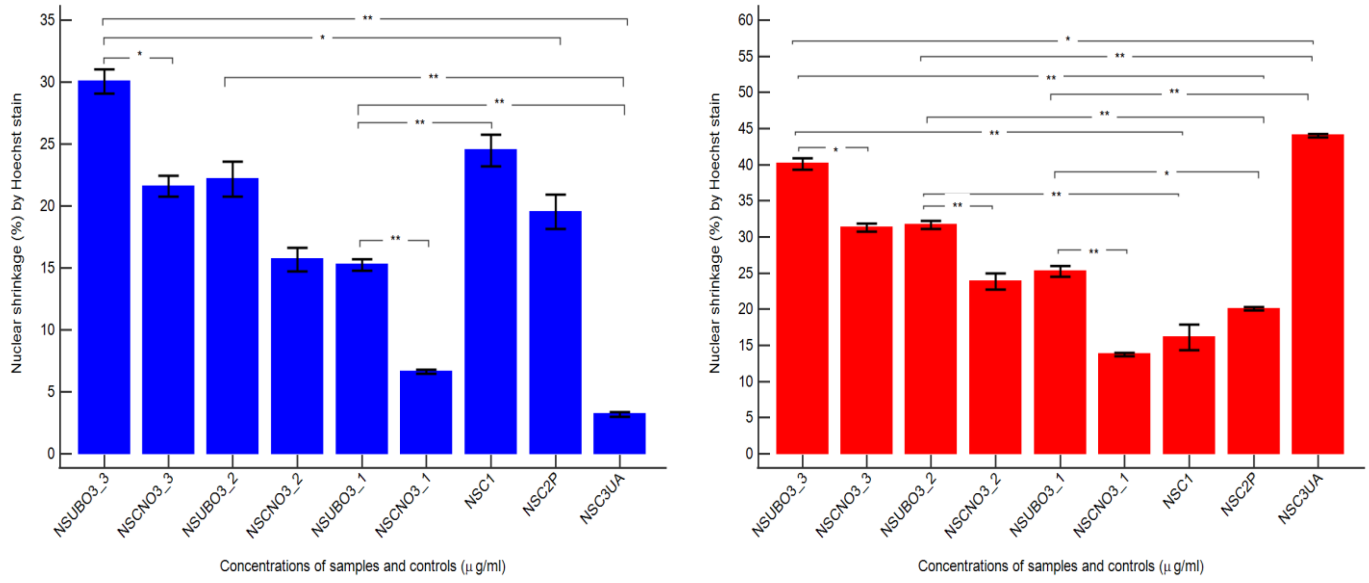

(a) **(b)**

Figure S5. Statistical analysis of nuclear shrinkage in blood cells (**a**) and CLS-354 tumor cells (**b**) * $p < 0.05$ together with ** $p \leq 0.01$ indicate statistically significant differences between controls and samples. C1—1% DMSO (negative control), C2P—5% Poloxamer 407 (positive control), C3UA—usnic acid (positive control), NS—nuclear shrinkage, A—autophagy. [3:3; 3:2; 3:1]—culture medium/sample ratio (*v*/*v*).

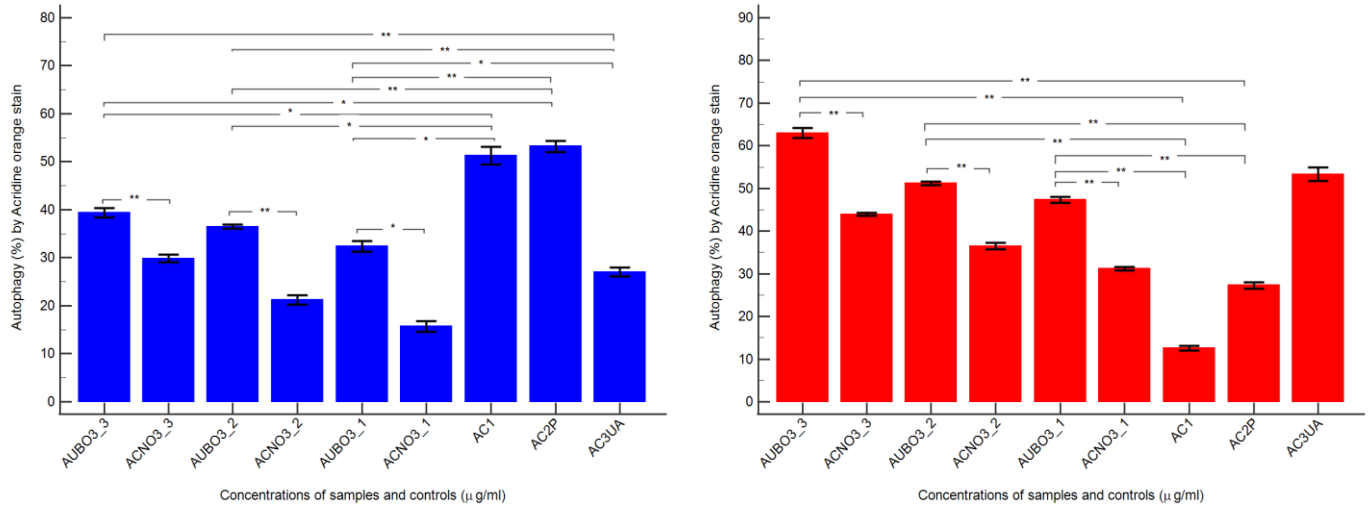

(a) **(b)**

Figure S6. Statistical analysis of autophagy in blood cells (**a**) and CLS-354 tumor cells (**b**).* $p < 0.05$ together with ** $p \leq 0.01$ indicate statistically significant differences between controls and samples; C1—1% DMSO (negative control), C2P—5% Poloxamer 407 (positive control), C3UA—usnic acid (positive control), A—Autophagy, NS—Nuclear Shrinkage, [3:3; 3:2; 3:1]—culture medium/ sample ratio (*v*/*v*)

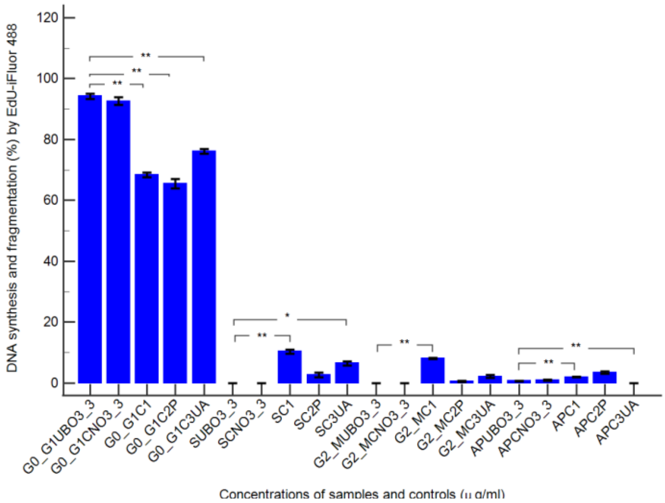

(a)

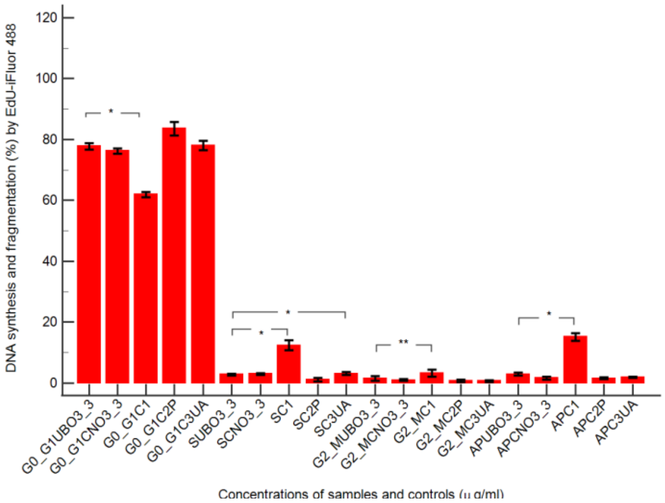

(b)

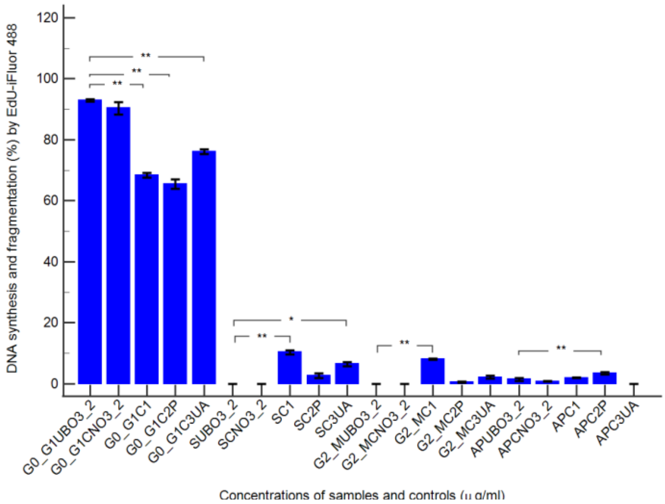

(c)

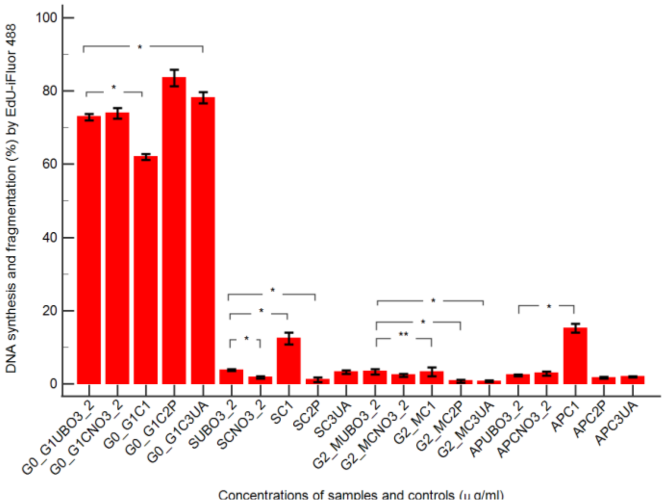

(d)

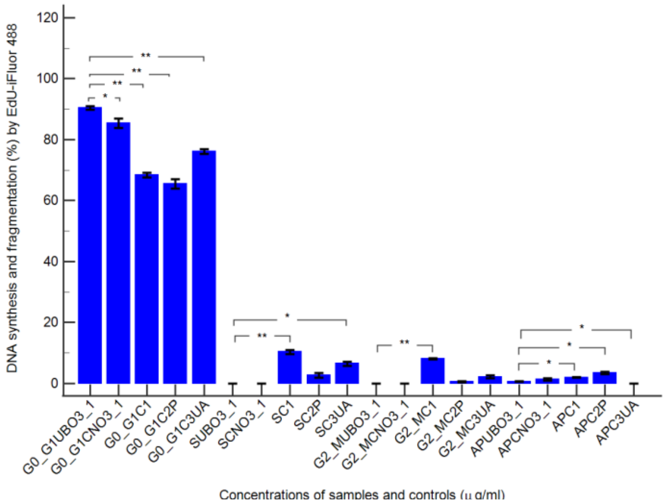

(e)

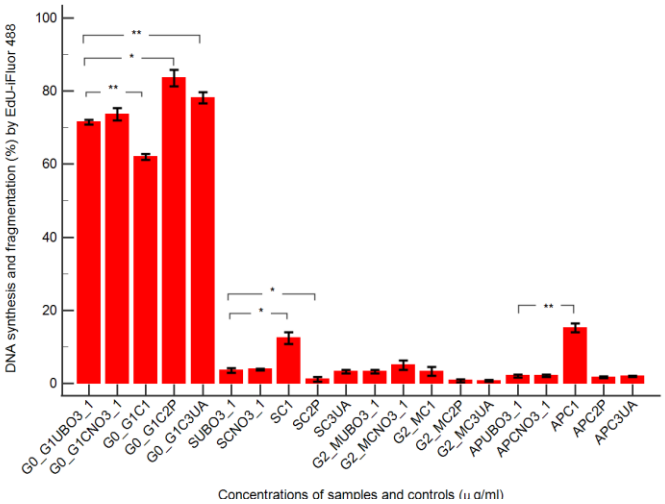

(f)

Figure S7. Statistical analysis of DNA synthesis and fragmentation in blood cells (**a, c, and e**) and CLS-354 tumor cells (**b, d,** and **f**) after 24 ore exposure to UBO and CNO in different concentrations: [3:3] (**a** and **b**), [3:2] (**c** and **d**) and [3:1] (**e** and **f**); $*p < 0.05$ together with $**p \leq 0.01$ indicate statistically

significant differences between control and samples made by paired samples *t*-test. C1—1% DMSO (negative control), C2P—5% Poloxamer 407 (positive control), C3UA—usnic acid (positive control), AP—cell apoptosis (DNA fragmentation, subG0/G1), S—DNA synthesis. [3:3; 3:2; 3:1]—culture medium/sample ratio (*v*/*v*).

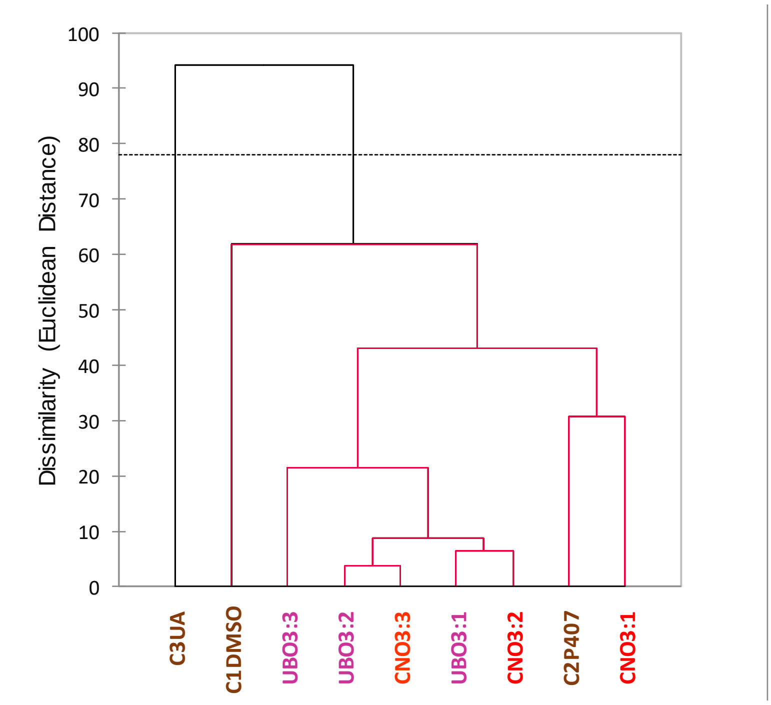

(e)

Figure S8. AHC-Dendrogram (**e**).

Table S1. Chemical constituents and minerals in UBO and CNO and their antiradical activity

Variable	UBO	CNO
pH	4	4
	Constituents	
Usnic acid (mg/g)	0.915 ± 0.018	0
TPC (mgPyE/g)	2.592 ± 0.097	2.243 ± 0.049
Al (µg/g)	7.688 ± 0.086	0.975 ± 0.049
Ca (µg/g)	76.818 ± 14.289	74.711 ± 4.048
Mg (µg/g)	6.951 ± 0.177	6.852 ± 0.099
Cr (µg/g)	0.195 ± 0.005	0.158 ± 0.002
Cu (µg/g)	0.155 ± 0.002	ND
Ni (µg/g)	0.713 ± 0.005	0.339 ± 0.004
	Antiradical activity	
% DPPH-radical scavenging	82.182 ± 0.595	64.806 ± 0.399
DPPH IC50 (mg/mL)	0.942 ± 0,004	1.361 ± 0,008

UBO—*U. barbata* extract in canola oil, CNO— canola oil, TPC—total phenolic content, mg PyE/g = mg Equivalents Pyrogallol/g
